# Association between Positivity and Smoking Cessation

**DOI:** 10.1155/2014/780146

**Published:** 2014-05-22

**Authors:** Maria Caterina Grassi, Guido Alessandri, Stefania Pasquariello, Michela Milioni, Domenico Enea, Mauro Ceccanti, Paolo Nencini, Gian Vittorio Caprara

**Affiliations:** ^1^Department of Physiology and Pharmacology, “V. Erspamer” School of Medicine, Sapienza University of Rome, Piazzale Aldo Moro 5, 00185 Rome, Italy; ^2^Department of Psychology, Faculty of Medicine and Psychology, Sapienza University of Rome, Via dei Marsi 78, 00185 Rome, Italy; ^3^Department of Clinical Medicine, Sapienza University of Rome, Viale dell'Università 37, 00185 Rome, Italy

## Abstract

The literature documents that personality characteristics are associated with healthy lifestyles, including smoking. Among positive traits, Positivity (POS), defined as a general disposition conducive to facing experience under a positive outlook has shown robust associations with psychological health. Thus, the present study investigated the extent to which POS is able to predict (i) relapse after quitting smoking and (ii) the desire to smoke again. All participants (481) had previously attended a Group Counselling Program (GCP) for Smoking Cessation (from 2005 through 2010). They were contacted through telephone interview. Among participants, 244 were ex-smokers (age: years 56.3 ± 10.08, 52% female) and 237 were still-smokers (age: years 55.0 ± 9.63; 63.5% female). The association of POS with “craving to smoke” levels was assessed with multivariate linear regression analysis while controlling also for important differences in personality such as conscientiousness and general self-efficacy, as well as for gender and age. Results showed that POS was significantly and negatively associated with smoking status and with craving to smoke. Among covariates (i.e., conscientiousness, generalized self-efficacy), gender was associated with smoking status and with craving to smoke. Altogether these findings corroborate the idea that POS plays a significant role in sustaining individuals' efforts to quit smoking.

## 1. Introduction

In Italy about 11 million adults are smokers, 20.7% of the entire adult population, according to the Osservatorio Fumo, Alcol e Droga [1]. Mortality trends over time for men and women demonstrate that smoking is “a huge threat to public's health” and explicitly posits “cigarette smoking among the most important health hazard” [2, 3]. As it stands, smokers lose at least one decade of life expectancy, as compared with those who have never smoked. Likewise, for people who smoke, the risk of death from cigarette smoking continues to increase over the years. Hence it has been established that smoking killed about 100 million people in the 20th century and it will kill about 1 billion in the 21st century [2, 3]. Thus, identifying reliable psychological predictors of smoking cessation seems a noteworthy enterprise.

In this regard, a large body of research has recently focused on human strengths and personality qualities associated with mental and physical health [4], with an emphasis on the personality characteristics associated with healthy lifestyles, including smoking [5–7]. Empirical studies on tobacco dependence have reported positive associations between quality of affective experience and the status of nonsmokers, reporting that nonsmokers experience higher quality of their life than smokers [8, 9]. Recently, Fidler and West [10] described “the enjoyment to smoke” as an important predictor of the individual's engagement in quitting smoking. These findings are a bit surprising as they seem not to be consistent with other results showing that quitting smoking can lead to experiencing a deterioration in the perceived quality of life [11–13]. All in all, it is likely that a decrease in self-perceived quality of life may nonetheless occur when smokers believe that quitting smoking means to lose an important source of enjoyment [14, 15] and that this eventually has the potential for making them less happy [16]. In this regard, individual differences in personality may play a major role in determining the attraction of smoking and the ability of quitting smoking. Among personality variables that may help to address the various issues associated with happiness and smoking, Positivity (POS) [17–20], namely, a personality trait associated with an individual disposition to view oneself, life, and the future under a positive outlook, appears a good candidate.

### 1.1. Positivity (POS)

Theoretically, POS represents a basic disposition that pervasively affects how people view themselves and the world, colours their relations with other people, and shapes their expectations about the future [17, 19]. The theory of POS [17, 21, 22] suggests that the personality features assessed by this construct represent basic assets that exert fundamental biological functions. In this regard, Caprara and associates [17–19, 21, 22] suggested that people could not face the experiences of aging and death, nor cope with the adversities and losses of life, unless they are equipped with the basic belief that they are worthy of regard, that life is worth living, and that the future is promising. Positivity is conceptualized as a trait-like basic disposition [17, 22] identified with what is common to self-esteem, life satisfaction, and optimism. Findings from twin studies [20] have converged with longitudinal and cross-sectional data in attesting to the trait-like nature of POS and to its stability [21]. Cross-cultural studies have documented the generalizability of POS factorial structure across countries that differ widely in terms of cultural models of self, language, cultural and historical roots, and ways of life [18, 22]. Recent studies posited POS among the major predictors of health, quality of friendships, resiliency, and positive affectivity over an extended length of time in the transitions from adolescence to adulthood [21]. Finally, they attested a correlation of POS with success at work in samples of adults [23].

### 1.2. Study Aims

This is the first study where POS has been adopted in the field of drug addiction. It aims to investigate the extent to which POS is able to predict (i) relapse after quitting smoking and (ii) the desire to smoke again. In pursuing this aim, we were particularly solicited by the recent data on the incidence and consequences of smoking over time.

### 1.3. Gender, Age, Conscientiousness, and General Self-Efficacy as Potential Confounders

To make our results more compelling, in addition to examining the predictive value of POS, other important individual differences in personality such as conscientiousness and general self-efficacy beliefs have been taken into account together with gender and age. All these variables have been previously associated to health related behaviors [24] and to nicotine addiction [25–27]. Whereas gender and age are well-known sociodemographic covariates of smoke addiction [25–27], the mechanisms linking personality to smoke addiction deserve some more attention. People high in conscientiousness are more likely to enact specific conscientious behaviors, such as taking better care of one's health, which in turn lead to better health. As demonstrated by empirical studies a large part of taking care of one's health involves avoiding health-damaging behaviors, such as smoking [24]. Individuals high in general self-efficacy beliefs are expected to possess more robust coping strategies necessary to maintain smoking cessation. Over the years, a number of empirical studies have repeatedly shown that individuals higher in general self-efficacy show a fewer episodes of relapse after an initial treatment [28]. These covariates were considered also for methodological reasons. As stated by Wiggins [29] the demonstration of incremental validity against well-established measures (such as conscientiousness and general self-efficacy beliefs) and already known predictors (such as gender and age) is a basic step in the study of the relevance and of the utility of newly introduced psychological constructs, such as POS.

## 2. Methods

### 2.1. Baseline Characteristics of Participants before Entering the Group Counselling Program (GCP) for Smoking Cessation

From January 2005 to December 2010, 686 subjects motivated to quit smoking (292 males and 394 females), average age 49.9 (SD ± 10.7) years, and smoking an average of 22.7 (SD ± 9.5) cigarettes/day for a period of 32.7 (SD ± 11.1) years were recruited by the outpatient unit at the Teaching Hospital Umberto I, Policlinico of Rome, “Sapienza” University of Rome. All patients attended a 6-week Group Counselling Program (GCP) for Smoking Cessation [30–32] and, in the absence of specific medical problems, were asked if they wanted to add to counselling a pharmacologic therapy consisting of nicotine replacement therapy, or Bupropion for a seven-week period or Varenicline (starting in 2007) for a twelve-week period according to tobacco treatment guidelines [33, 34]. Pharmacological treatment was accepted by 321 (46.8%) of the participants. Prior to admission to the GCP for smoking cessation, subjects underwent a structured interview about their smoking history. The amount of exhaled carbon monoxide (CO; Smokerlyzer Monitor Bedfont Scientific Ltd., Rochester, England; cutoff: 10 ppm) was taken as a measure to confirm the subjects' current smoking status. The level of nicotine dependence was measured using both the Fagerström Test for Nicotine Dependence (FTND) [35] and the Severity of Dependence Scale (SDS) [36–39]. A self-efficacy test, whereby the subject had to rank himself or herself from 0 to 10 on the possibility of “becoming a nonsmoker” [30, 31, 40], was administered. During this interview, other parameters were collected, such as weight, body mass index (BMI), and the GCP was explained. Follow-up assessments to verify continuous abstinence rate were carried one year after the quit day; subjects were asked about their smoking status and invited to come to the hospital to complete the final form and to measure exhaled CO. Values of exhaled CO obtained from the 207 subjects who came to the 1-year follow-up visit were consistent with the self-reported smoking status (exhaled CO: 26.6 ppm among smokers, *n* = 23, and 1.9 ppm among nonsmokers, *n* = 184).

### 2.2. Telephone Interview and Ethical Approval

All data for the present study were gathered during a follow-up telephone interview, conducted 2–6 years after the end of the previous described GCP for smoking cessation (from January to December 2012). All subjects who attended the GCP were called. All interviews were conducted by a team of four expert licensed clinical psychologists. Each interviewer used structured protocol to conduct the interview and filled out a prestructured paper questionnaire with answers obtained by subjects (questionnaires are available upon request).

In particular, besides the measures of interest described below, subjects were asked about their smoking behavior, since their last follow-up, with the following questions: “Do you smoke at the moment?” If the answer was “no,” this second question was posed: “How long you have not being smoking: years, months, days?” whereas if the answer was “yes,” they were asked: “How many cigarettes do you smoke daily/weekly?”

The study was approved by the local ethical committee, and each participant approved informed consent before all questions were submitted through the telephone interview.

### 2.3. Measures of Interest

*Smoking Status.* Subjects were asked about their smoking status. Their answers were coded “0” (ex-smokers) or “1” (still-smokers), depending on their status.

*Craving to Smoke*. Subjects were asked to fill in a Visual Analogue Scale (VAS) to measure the intensity of their “craving to smoke” [41, 42].* Positivity* (POS). To measure POS we used the P-Scale [43]. The scale is composed by eight items, of which seven are positively worded (e.g., “I feel I have many things to be proud of”), and one was negatively worded (e.g., “At times, the future seems unclear to me”). This item was reverse scored as appropriate, to indicate high positivity. Participants were asked to provide their ratings using a 5-point scale ranging from 1 (strongly disagree) to 5 (strongly agree). The individual score on POS is computed as the individual mean score on the eight items of the P-Scale. Cronbach's alpha of the eight-item scale was .78.

*Generalized Self-Efficacy (GSE)*. The GSE was measured using three items of the original version of the scale developed by Schwarzer and Jerusalem [44] in Germany and then translated into many languages. This scale was designed to assess a general sense of perceived self-efficacy used to cope with a variety of demands in life. Participants were asked to provide their ratings using a 5-point scale ranging from 1 (strongly disagree) to 5 (strongly agree). Cronbach's alpha was .72.

*Conscientiousness (CONSC)*. Participants rated their conscientiousness as personality trait on 4 items derived by the Big-Five Questionnaire [45]. Participants rated their orderliness, precision, and the fulfilling of commitments using a 5-point Likert scale (1 = strongly disagree to 5 = strongly agree). Item example was “usually, when I finish a work, I check the accuracy of every detail.” Cronbach's alpha was .75.

### 2.4. Statistical Analysis

Before analyses, the normality assumption was checked and accepted for all continuous variables, by looking at coefficients for skewness and kurtosis. These coefficients were all below recommended standards of 1.00 [46]. In detail, coefficients for skewness ranged from −.58 (POS) to .86 (GSE), and coefficients for kurtosis ranged from .16 to .43 (CONSC). Statistical comparisons between groups for continuous variables were performed using two sample *t*-tests, whereas categorical variables were analyzed using Pearson's chi square tests. Correlations were computed using tetrachoric coefficients for couples of dichotomous variables (i.e., smoking status and gender), and poliserial coefficients for couples of continuous (i.e., scores on the P-Scale, GSE, and CONSC) and dichotomous variables. Statistical analyses were performed using SPSS 20.0. To evaluate the role of variables related to the natural history of smoking as predictors of smoking cessation, one logistic regression analysis was carried out with smoking status as the outcome (nonsmokers versus still-smokers). The association of POS with “craving to smoke” levels was assessed with multivariate linear regression analysis. All regression equations were adjusted for CONSC, GSE, gender, and age in order to evaluate if individual's level of POS was independently associated with each of the above outcomes (i.e., smoking status and craving to smoke). The adequacy of the logistic regression equation was investigated using the Hosmer-Lemeshow (H-L) test, which indicates the extent to which the model provides better fit than a null model with no predictors. If the H-L goodness-of-fit test statistic is greater than .05, one fails to reject the null hypothesis that there is no difference between observed and model-predicted values, implying that the model's estimates fit the data at an acceptable level. We also reported the Nagelkerke* R*-square as a measure of the total amount of variability in the dependent variable explained by the predictors considered in the equation. The adequacy of the linear regression equation was investigated using the* R*-square coefficient, which indicates how well data points fit a statistical model. A significant* R*-square coefficient indicates that the proportion of variance explained by a regression equation is greater than that explained by a model with no predictors and thus is worth of empirical consideration.

## 3. Results

**Figure 1:**
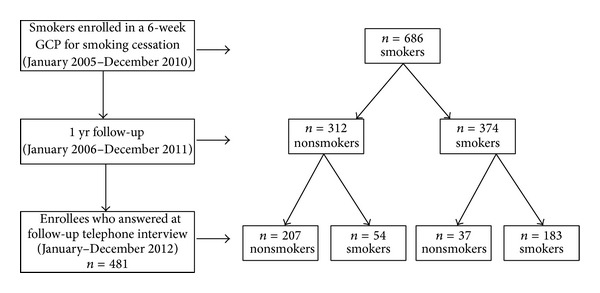
Chart of the subjects who previously attended a 6-week Group Counselling Program (GCP) for Smoking Cessation and answered at the follow-up telephone interview.

In the present study all the 686 subjects, who attended the GCP and who were nonsmokers (*n* = 312, 45%) or smokers (*n* = 374, 55%) at previous 1-yr follow-up, were contacted through a telephone call, to ascertain the present smoking status (Figure 1). Seventy percent (*n* = 481) of the subjects answered at the telephone call while thirty percent (*n* = 205) resulted unreachable (no answered, *n* = 144), or refused the interview (*n* = 43), or were deceased (*n* = 18).  Table 1 shows the baseline characteristics and smoking history of the 481 subjects at enrolment, before entering the six-week GCP for smoking cessation. At enrolment, according to the results at the telephone interview, ex-smokers compared to still-smokers showed lower values of (1) FTND (5.2 ± 2.1 versus 5.6 ± 2.1, *P* < .05), (2) SDS (9.6 ± 2.4 versus 10.1 ± 2.2, *P* < .05), (3) exhaled CO (22.1 ± 11.7 versus 24.5 ± 13.4, *P* < .05), (4) BMI (24.4 ± 3.8 versus 25.2 ± 4.0, *P* < .05), and (5) were less suffering from respiratory pathologies (73.4% versus 81.8%, *P* < .01). Moreover Table 2 shows the “current” characteristic of the same subjects according to their smoking status at the telephone interview: actually ex-smokers or still-smokers. More in detail, ex-smokers and still-smokers differed (*P* < .05) in (1) gender (ex-smokers were more likely to be males than females), (2) body weight (still-smokers weighted less), (3) weight gain (ex-smokers were more likely to gain weight), (4) craving to smoke (still-smokers reported higher levels of VAS), and (5) quit attempts other than the GCP (more attempts for still-smokers). With regard to psychological variables, we found statistically significant differences (*P* < .05) only for POS: ex-smokers reported higher levels of POS.

Table 3 contains also the zero-order correlations between the major study variables (gender, age, POS, GSE, CONSC, smoking status, and craving to smoke). These correlations represent the first-order effects of each variable without controlling for the effect of the others. Results showed that male gender was inversely associated with both smoking status and craving to smoke, but positively related to POS. Age was negatively related with POS, but positively related with generalized self-efficacy and conscientiousness. POS was negatively related to both smoking status and craving to smoke. Of interest, the three personality traits were positively associated with each other, and craving to smoke was positively and significantly associated with smoking status. With regard to sociodemographic variables, females referred to smoke and craving to smoke more than males. Males reported higher scores in POS than females. Younger individuals reported higher scores in POS than older individuals. Finally, older individuals showed higher scores in GSE and in CONSC than younger. Importantly, individuals higher in POS seemed more incline to quit smoking and to crave less to smoke than individual low in POS.

### 3.1. The Predictive Value of POS

Table 4 shows results from multiple logistic and linear regression analyses. POS resulted significantly (*P* < .05) and negatively associated with smoking status (i.e., more positive individuals were more likely to be ex-smokers) and with craving to smoke (i.e., more positive individuals referred lower levels of craving to smoke). Among covariates, only gender was associated with smoking status (i.e., females were more likely to be still-smokers) and with craving to smoke (i.e., females referred higher levels of craving to smoke). Age, CONSC, and GSE beliefs were not associated with the outcomes considered. All models showed an adequate data fit, as attested by nonsignificant H-L test and significant* R*-square values (Table 4).

## 4. Discussion

Identification of positive traits, behaviors, emotions, and cognitions that may promote well-being and flourishing has become a major goal of recent psychological research. Indeed, psychological literature is increasingly recognizing and appreciating the value of individual's characteristics and qualities as crucial elements for a healthy and long life [5–8]. This is reflected in the novel orientation of psychological science in promoting empirical studies aimed to identify ways able to lead individuals to pursue and maintaining healthy life habits [15, 16]. This study contributed to this literature by presenting innovative data corroborating the value of POS, a positive psychological trait, as a predictor of smoking cessation.

All in all, present findings are consistent with the idea that POS may play a moderate (as attested by the* R*-square values), although non-negligible role in sustaining individuals' efforts to quit smoking. In accordance with our hypothesis, levels of POS positively predicted smoking status, with more positive individuals more likely to be in the ex-smokers conditions. Of relevance our findings suggest that POS not only predicts smoking status, but also reduces the craving to smoke. These results are of interest, since they underline the potential represented by this newly introduced positive personality trait. As it stands, positive ex-smokers were characterized by a lower desire to revert to the past negative habits.

While the benefits associated with POS for smoking cessation are clearly attested by our findings, our data are mute with respect to the psychological mechanisms processes through which POS translates into this healthier lifestyle (i.e., nonsmoking). Since this is the first study that examines the association with POS and smoking cessation, it seems premature to present hypotheses in this regard. Speculations are however possible, although limited.

We are inclined to think that POS may act as a motivational mechanism that sustains individual's efforts in quitting a bad habit (such as smoking), by leading them to a favourable evaluation of the efforts done. It is unlikely that also ex-smokers have not had recidivisms, nor have they never been tempted by a friend who smoked, nor have they never been close to restart with the bad habit. In our view, positive ex-smokers are sustained in their walk out from smoking by positive feelings about their ability to resist, and, if at times they may have relapsed, they tend to evaluate their recidivism as an “incident,” or also a “momentary distraction.”

On a related side, it is likely that POS may contribute to quitting smoke by fostering high tolerance to stress, resiliency, and commitment to valued goals, such as quitting smoke [23]. A certain amount of distress is normal when people try to quit a bad and pervasive habit, such as smoking. But some people may be affected more than others. Moreover, daily stressful events may trigger the recurrence to smoking as a previously experienced successful coping strategy.

By leading individuals to see events as predictable and generally occurring in one's best interest [17, 18, 22], POS may lead people to perceive events in their life as less threatening, their life and health related goals as more attainable, and to reduce the impact of the challenges and stressors resulting from daily experiences and social interactions. Thus POS may help to prevent the pernicious effect of stress in guiding individuals toward previous bad habits.

Despite speculations, understanding the psychological pathways, through which POS sustains healthy habits, represents a critical point that should find an answer in future studies. The knowledge of these mechanisms may indeed likely lead to more effective psychological interventions.

## 5. Conclusions

Looking on POS as a predisposition opens new avenues to both research and practice concerned with promoting human potentials and strengths. Whereas recent findings suggest that POS, although stable, is malleable to change [17, 18, 22], both whether and how POS has a beneficial function, and whether and why a lack or an excess of POS may carry negative consequences, deserve further investigation. From our perspective, such knowledge is crucial to individuate practices useful to effectively promote and sustain individuals' POS. Likely, the same principles that have proved to foster smoking cessation through mastery experiences may serve to nurture positivity through mastery experiences in the domain of emotion regulation and interpersonal relations [40]. Likely, the more the people are able to manage their emotions and to benefit from their relations, the more they have reason to be confident in themselves and to look on life and on the future under a positive outlook and the more they are able to accord their habits to healthy styles [17, 22].

On a related side, a better understanding of the biological correlates of POS may be useful to fully clarify how to use POS for promoting valued changes in individuals' lifestyles. More broadly, the knowledge of the biological substrates and of the pathways of influence that link POS with other constructs associated with psychological well-being (e.g., positive affect) may enhance our comprehension of the complex interplay between the biological and psychological systems in promoting and sustaining health.

## 6. Limitations

We acknowledge a few limitations of the present contribution. Although POS relies on a set of subjective evaluations that are not easily accessible other than self-reports, other methods such as implicit measures, clinical interviews, and reports from other informants would be useful complements to the use of self-report data. Moreover, the cross-sectional nature of these data does not permit strong inferences about causal effects. Notwithstanding these limitations, the results contribute to the understanding of the relation between POS and positive affectivity.

## Figures and Tables

**Table 1:** Baseline characteristics and smoking history of participants who answered at the telephone call, at enrollment, from January 1, 2005 through December 31, 2010, before entering the six-week Group Counselling Program (GCP) for Smoking Cessation.

Characteristics	*n* (%) or mean ± SD *n* tot = 481
Females (%)	283 (58.8)
Age: years (range)	50.9 ± 9.9 (24–74)
Years of smoking (range)	33.6 ± 10.4 (2–62)
Education (%)	
Primary school	68 (14.1)
Middle school	235 (48.9)
Degree	178 (37.0)
Occupation (%)	
Unemployed/household	47 (9.8)
Employed or students	351 (73.0)
Retired	83 (17.2)
Marital status (%)	
Single	98 (20.4)
Married/living together	271 (56.3)
Divorced or separated or widowed	112 (23.3)
Family history of smoking: yes (%)	430 (89.4)
Others smokers in household: yes (%)	201 (41.8)
Body weight: Kg	69.8 ± 13.6
BMI (Kg/m^2^)	24.8 ± 3.9
Exhaled carbon monoxide (CO) (ppm)	23.3 ± 12.6
Number of cigarettes per day	22.6 ± 9.3
Number of previous quit attempts (%)	
0	76 (15.8)
1	129 (26.8)
2	122(25.4)
3+	154 (32.0)
Number of cups of coffee per day	3.3 ± 1.8
No alcohol consumption	107 (22.2)
Fagerström Test For Nicotine Dependence (0–10)	5.4 ± 2.1
Severity of Dependence Scale (0–15)	9.9 ± 2.4
Craving Scale (0–100)	61.2 ± 20.2
Self-efficacy evaluation (0–10)	5.8 ± 2.2
Respiratory pathologies: yes (%)	373 (77.5)
Cardiovascular diseases: yes (%)	288 (59.9)

**Table 2:** Characteristics of the 481 participants enrolled in the study, according to their smoking status at the telephone interview.

	Ex-smokers	Still-smokers	*P* value*
Number of subjects	244	237	
Age: years	56.3 ± 10.1	55.0 ± 9.7	.121
Females (%)	52.0	65.8	.001
Occupation (%)			
Unemployed/household	8.6	7.6	.675
Employed or students	60.7	64.6	
Retired	30.7	27.8	
Marital Status (%)			
Single	17.0	19.9	.005
Married/living together	65.1	51.3	
Divorced or separated or widowed	17.9	28.8	
Body weight: Kg	74.6 ± 14.8	67.5 ± 13.2	<.001
Weight gain from enrollment (Kg)	2.8 ± 6.8	−0.2 ± 6.6	<.001
Children at home: yes (%)	25.0	19.1	.079
Number of cigarettes per day	0	17.9 ± 11.6	
Number of further quit attempts (%)			
0	79.2	55.6	<.001
1	14.4	23.1	
2	3.8	11.5	
3+	2.5	9.8	
Takes prescription drugs: yes (%)	64.3	59.5	.158
Craving Scale (0–100)	7.8 ± 19.1	69.2 ± 27.8	<.001
POS	3.8 ± 0.7	3.6 ± 0.8	.016
GSE	4.0 ± 0.6	3.9 ± 0.7	.241
CONSC	4.2 ± 0.6	4.1 ± 0.6	.503

*Note*. Data are reported as mean ± SD or as percentage of the total number of subjects observed for each group. POS: Positivity Scale; GSE: General Self-Efficacy Scale; CONSC: Conscientiousness Scale. *Pearson's chi square or Student's *t*-test.

**Table 3:** Zero-order correlations among gender, age, conscientiousness, generalized self-efficacy, Positivity, smoking status, and craving to smoke (*n* = 481).

	Gender	Age	POS	GSE	CONSC	Smoking status at interview	Craving to smoke
Gender	1						
Age	.03	1					
POS	.10*	−.12**	1				
GSE	.01	.10*	.42**	1			
CONSC	−.03	.10*	.37**	.49**	1		
Smoking status at interview	−.14*	−.07	−.11*	−.05	−.03	1	
Craving to smoke	−.12*	−.06	−.18**	−.08	−.04	.79**	1

*Note*. POS: Positivity; GSE: Generalized Self-Efficacy; CONSC: Conscientiousness.

*P < .05 and **P < .01. Correlations were computed using tetrachoric coefficients for couples of dichotomous variables (i.e., smoking status and gender), poliserial coefficients for couples of continuous and dichotomous variables (i.e., GSE and gender), and Pearson's coefficients for continuous variables (i.e., scores on the POS, GSE, and CONSC; smoking status at interview: “*still-smokers* or *nonsmokers* at the telephone interview”).

**Table 4:** Results from multiple regression analyses (*n* = 481).

	Smoking status at telephone interview^a^				
	Beta	Wald	*P* value	OR	95% CI
Gender	−.53	7.77	.00	.59	.40, .85
Age	−.02	3.14	.08	.98	.96, 1.00
CONSC	.07	.15	.69	1.07	.75, 1.53
GSE	−.01	.01	.92	.98	.71, 1.36
POS	−.31	4.61	.03	.73	.55, .97

Test of Hosmer-Lemeshow = *χ* ^2^(8) = 12.56, *P* = .13. *R*-square = .04					

	Craving to smoke				
	Beta	*t*-test	*P* value	—	95% CI

Gender	−.10	−2.14	.03	—	−14.7, −.64
Age	−.09	−1.88	.06	—	−.70, .02
CONSC	.04	.82	.41	—	−3.88. 9.47
GSE	−.01	−.11	−.92	—	−6.38, 5.73
POS	−.19	3.75	.00	—	−15.5, −4.84

R-square = .06, P = .041					

^a^Referent class: *still-smokers* at the telephone interview; gender coded: 0: females, 1: males; OR: odds ratio.
